# Off-Label Treatment in Inflammatory Skin Diseases—European Point of View

**DOI:** 10.3390/jcm14072376

**Published:** 2025-03-30

**Authors:** Julia Sternicka, Roman J. Nowicki, Leszek Bieniaszewski, Dorota Purzycka-Bohdan

**Affiliations:** 1 Department of Dermatology, Venereology and Allergology, Medical University of Gdańsk, University Clinical Centre, 80-214 Gdańsk, Poland; roman.nowicki@gumed.edu.pl; 2 Clinical Physiology Unit, Medical Simulation Centre, Medical University of Gdańsk, 80-204 Gdańsk, Poland; leszek.bieniaszewski@gumed.edu.pl

**Keywords:** off-label, pharmacotherapy, atopic dermatitis, psoriasis, acne vulgaris, rosacea

## Abstract

Off-label treatment is the use of a drug approved for marketing, outside the registration in terms of indication, age group, dose or route of administration. Despite the constant appearance of new preparations on the market, treatment outside the SmPCs guidelines is a current clinical problem. It is believed that it is based on the needs of patients unmet by classical therapy methods. This work focuses on off-label treatment in inflammatory dermatoses such as atopic dermatitis, psoriasis, acne vulgaris and rosacea. Publications on this subject, available on PubMed, Google Scholar and the Cochrane Library, were analyzed in the form of a review, taking into account the mechanisms of action, efficacy and safety of preparations. Based on the literature analysis, it can be concluded that the use of drugs outside the SmPC indications is a common situation in dermatology. However, it is difficult to determine its exact frequency—there is a lack of data on the prevalence of off-label appliances in inflammatory dermatoses from a European perspective. Publications demonstrate varying effectiveness and safety of this form of therapy, depending on the specific preparation. Off-label treatment in dermatology remains an important and current clinical issue that should be explored in further research.

## 1. Introduction

Off-label treatment is a phenomenon that has been gaining increased attention in recent years [1,2]. To properly understand that concept, it is essential to consider its meaning. Off-label therapy is a term applied to describe the use of a drug registered in a given country that goes beyond the guidelines in the Summary of Product Characteristics (SmPC) [1,2,3,4,5,6]. That includes usage in a different indication, age group, dose, route of administration and pharmaceutical form [1,3,6]. It is necessary to distinguish this method of treatment from an unlicensed one and, above all, from illegal, improper or contraindicated use of a drug [3,4,5,7,8]. In order to apply the off-label procedure, the selected medication must be registered in a given country [3,4,5,9]. That is not possible without the consent of the appropriate licensing authority, such as the European Medicines Agencies (EMA), the Food and Drug Administration (FDA) or national drug agencies [3,4,7]. The process of authorizing the marketing of a given drug is subject to many restrictions, including high requirements regarding the safety and quality of preparations [4,7,10]. There must also be a scientific basis for off-label usage; for example, publications must report the effectiveness of a given drug in patients with a selected disease [7,11,12]. Despite that, as a consequence of the absence of registration, there is usually a lack of appropriate, large-scale studies, such as randomized clinical trials (RCTs) in specific patient groups [1,7,13]. So, why is this form of therapy in use? It is believed that by going beyond the framework defined by SmPCs, it is possible to fulfill the needs of patients that have not been met by standard treatment [2,3]. This form of therapy is particularly often applied in people with rare diseases, but also in cases resistant to standard therapies registered in a given entity [1,3,11,14]. Groups of patients, who are more usually treated outside the guidelines of SmPCs, are also children and pregnant women, which is related to the limited number of preparations tested in these populations and thus registered for use in these individuals [1,7,13,14,15,16,17,18].

As more drugs appear, one would expect off-label use to decrease, yet this sort of treatment remains prevalent even with the passage of time [6,19]. Regardless of the arrival of new drugs on the market, not all patient needs are met [2,6]. Despite this, companies do not often decide to expand the registration of available drugs [6,15]. Reports from the European Committee (EC) suggest that this may be due to factors such as the lengthy and costly marketing authorization process for new registrations, shortage of funds, insufficient motivation to expand registration of approved drugs and lack of expected reimbursement [3]. As a result, some groups of patients are unable to achieve a satisfactory therapeutic effect with typical treatments [3]. This does not go unnoticed by the medical community and is reflected in legal regulations, both national and wide-reaching, e.g., European directives, where attempts are made to control this phenomenon so that the treatment is effective and, above all, safe for the patients in need [3].

One of the areas of medicine in which off-label use of drugs is not an uncommon phenomenon is dermatology [6,20,21,22]. Skin conditions that sometimes require this non-standard approach include atopic dermatitis, psoriasis, rosacea, acne vulgaris as well as many other, less prevalent dermatoses [21,22,23,24]. The aim of this work is to summarize the current state of knowledge on off-label treatment of the listed diseases, both in terms of implemented drugs with their mechanisms of action, efficacy and safety. To achieve this, literature research was conducted using PubMed, Google Scholar and the Cochrane Library. The following terms were used for the search: “off-label”, “dermatology”, “atopic dermatitis”, “psoriasis”, “acne vulgaris”, “rosacea”. Articles in Polish and/or English, containing the above-mentioned key words were analyzed. The exclusion criteria included non-indexation in the specified databases (PubMed, Google Scholar and the Cochrane Library), unclear source, lack of full-text availability and publications in languages other than Polish or English. Documents made available by the European Medicines Agencies (EMA), Food and Drug Administration (FDA), European Committee (EC) and Summaries of Product Characteristics (SmPCs) were also taken into consideration.

## 2. Atopic Dermatitis

Atopic dermatitis (AD) is a widespread chronic dermatosis [25,26]. The likelihood of developing AD during one’s lifetime ranges from 10 to 30%, with the disease most frequently affecting children [25,27]. The pathogenesis of this disease is complex: immunological, genetic and environmental factors participate in its development [26,27,28,29,30,31,32,33]. Diagnostic basis for this dermatose has been defined, among others, in the Hanifin and Rajka criteria, among which the most characteristic symptom is considered to be pruritus [25,27,28,34]. This manifestation can be extremely troublesome for patients, which is associated with limitation of life activities and a decrease in their quality of life [27,29]. To assess the severity of AD, one can use for example the SCORAD index, which assesses the percentage of skin affected in the patient, the intensity of dermatological signs and the intensity of the patient’s subjective symptoms [35]. Thanks to this, a specific form of therapy can be recommended more consciously, depending on the severity of AD.

Depending on the SCORAD score assessment, patients can be offered emollients, which are the basis of treatment, local and/or systemic treatment [36]. Topically applied drugs typically involve anti-inflammatory drugs—mainly steroids, as well as topical calcineurin inhibitors (TCI) and others like crisaborole ointment [26,37,38,39]. Systemic forms of therapy should be used, when managing a severe form of AD, when appropriate topical treatment does not lead to clinical improvement, and when, despite treatment, the patient is unable to participate in normal daily activities [40]. They include oral glucocorticosteroids (GCSs), antihistamines of first and second generation as well as cyclosporine A (CsA), methotrexate (MTX), azathioprine (AZA), mycophenolate mofetil (MMF), biological drugs such as dupilumab, tralokinumab, lebrikizumab and Janus kinase (JAK) inhibitors: upadacitinib and abrocitinib [32,37,38,41]. Not all of these drugs are registered for use in AD and they can only be used in specific conditions; e.g., in specific age groups of patients (Table 1) [42]. The group of patients most frequently treated with drugs outside the SmPCs guidelines are children [42,43]. In 2015, it was estimated that up to 60% of patients with AD under 18 years of age may be treated that way [42].

Among the topical drugs used off-label in minors, scientific papers mention topical corticosteroids and TCI (tacrolimus, pimecrolimus) [20,44]. Most topical steroid drugs are approved for use in patients from 1 year of age (including alclometasone 0,05%, fluticasone 0.05%) or from 2 years of age (for example methylprednisolone 0.1%). GCS apply their immunosuppressive and anti-inflammatory effects by regulating the function of pro-inflammatory genes and cells, acting equally well in patients with a predominance of Th2 and Th1 lymphocytes [26]. This is achieved through the direct action of the steroid–receptor complex on the sensitive elements of the nucleus, which leads to changes in transcription and, consequently, protein synthesis [26].

Tacrolimus ointment at a concentration of 0.03% is registered for use from 2 years of age, and 0.1% from 16 years of age [45]. According to some publications, it is the most commonly used off-label medication in dermatology in the pediatric population, constituting over 20% of drugs outside the SmPCs [41]. In the case of pimecrolimus cream, there has been a significant change recently. This drug, previously registered for use in patients from 2 years of age, can currently be used on-label from 3 months of age [46,47]. TCIs, as their name suggests, act by inhibiting calcineurin, which prevents the activation of T lymphocytes and, consequently, the production of pro-inflammatory cytokines [26,45]. Both groups of locally applied drugs, GCS and TCI, are effective and have a good safety profile [45,48]. They constitute the basic, apart from emollient therapy, method of treating AD [37,38,49]. Therefore, conducting therapy that applies to SmPC guidelines in children with AD, who have not reached the age of 2, may constitute a significant therapeutic challenge and often requires the off-label use of drugs [50,51,52]. Therefore, it is necessary to provide detailed information to the parents of patients, who are often concerned about the safety of medications used in these age groups.

Systemic treatments can also be implemented in an off-label manner [53]. This group primarily includes anti-inflammatory drugs azathioprine, methotrexate and mycophenolate mofetil [42,54]. Until recently, the only registered medications for systemic therapy in AD were cyclosporine A and oral steroids [42]. However, it should be remembered that cyclosporine is not registered for all age groups and if used to treat minors, it would be outside the SmPCs guidelines [42]. Currently, dermatologists have an increasingly wide range of modern drugs at their disposal—Janus kinase inhibitors and biological drugs [55,56,57]. Thanks to this, the role of classic anti-inflammatory medications has decreased; nevertheless, they still play significant part in treatment of AD [53].

Cyclosporine (CsA) is registered for use in non-transplantation indications from 18 years of age [58,59]. By blocking calcineurin, CsA has an immunomodulatory effect on T lymphocytes, thereby calming the inflammatory response [45,60]. The use of CsA leads to a reduction in the severity of symptoms, both in children and adults [45,61]. However, application of this drug may lead to the development of hypertension, as well as the deterioration of renal parameters [62]. For this reason, CsA is usually a short-term option (3–6 months), because the risk of side effects increases with longer-term use [30,47,62].

Methotrexate (MTX), even with not being registered for the treatment of AD, is one of the most commonly used systemic drugs in this condition [63]. It acts by blocking the synthesis of ribonucleic acids, which leads to the inhibition of T lymphocyte function—one of the main axes of the development of AD [45]. Thanks to its long presence on the market, this drug has a well-defined profile, is cheap and also easily available [54,64]. It is long-term effective and is usually well tolerated [45,63,65]. Studies have shown a good safety profile of this drug even in children under 4 years of age, with the most popular side-effect being gastrointestinal ailment [66]. Nonetheless, it should be remembered that MTX has myelotoxic, hepatotoxic and teratogenic potential, which is why its use in women of reproductive age should be complemented by an effective form of contraception [62].

Mycophenolate mofetil is another systemic agent that can be used in the form of off-label therapy in the case of AD that does not respond to standard treatment [67]. Studies to date have shown the effectiveness of this drug in the treatment of the discussed dermatosis [67]. However, it can lead to complications, such as nephrotoxicity or malignancies [45]. It should therefore be used with caution, taking into account the ratio of benefits and losses in a specific patient, especially in the case of off-label use in minors [45].

Azathioprine (AZA) is an anti-inflammatory drug that leads to impaired DNA synthesis and thus lymphocyte proliferation, which reduces the inflammatory response. Although previous studies have shown the effectiveness of AZA treatment, it was associated with such severe side effects that a significant number of patients discontinued treatment with this drug [45]. These include vomiting, nausea and headaches. For this reason, implementing AZA in treatment of AD should be careful and well thought-out [45].

An extremely popular group of drugs applied in AD is antihistamines. Nonetheless, available preparations are not registered for this indication: according to the SmPC, most of them can be used in the case of urticaria and allergic rhinitis. Hydroxyzine is the only substance from this group, for which registration is extended to the treatment of pruritus. The validity of using antihistamines in AD is a matter of debate at the moment, but there is no evidence to support their routine implementation [68]. However, these are certainly safe drugs with a long history of use, so they are traditionally applied nonetheless [69].

Montelukast, a drug registered for the treatment of allergic rhinitis and asthma, is also sometimes used outside these indications. This leukotriene receptor antagonist, according to most available publications, is effective in managing AD, regardless of the age of patients [70]. However, there are also trials according to which the efficacy of montelukast is comparable to a placebo [71]. A publication by Kristina R Jamalyan et al. reported that adding montelukast to antihistamine therapy can significantly increase its effectiveness, which may be a new direction for the use of these drugs in the coming years [72]. Scientific evidence indicates the safety of this form of therapy and limited side effects [70].

Another group of drugs registered in AD, but not in every age group, are biologics and janus kinase inhibitors. The first group includes dupilumab, the first biologic drug registered for the treatment of AD, tralokinumab and lebrikizumab [73].

Dupilumab is a monoclonal antibody that blocks the IL-4 receptor. [55,73]. This impairs the inflammatory response, mainly the IL-4/IL-13 pathway, which plays a significant role in the pathogenesis of AD [41,55,71,74]. Clinical trials have shown its safety and effectiveness, not only in reducing visible skin lesions, but also in terms of pruritus and depressive mood disorders [56,73,75]. Nonetheless, it is not a drug without flaws. Problems that can be encountered in patients using dupilumab include incomplete remission of lesions and side effects [73]. The most common of these are conjunctivitis and local reactions at the injection site [41,55,56,57]. According to the SmPC, it can be used in patients over 6 months of age; however, until recently this drug was registered for use in people 12 years of age and older, which is why it often appears in the literature in the context of off-label use [32,56,76,77]. Currently, its use outside the SmPC guidelines has been significantly limited.

Lebrikizumab is a monoclonal antibody that blocks the activity of free IL-13 by binding to it, thus reducing the inflammatory response and preventing the impairment of the filaggrin gene expression, the proper function of which is the basis of a healthy epidermal barrier [26,55,73]. Clinical trials have shown the superiority of lebrikizumab in terms of efficacy not only over placebos, but also over topical steroids, which have had a well-established position in the treatment of AD for many years and constitute its basic element [73]. According to the SmPC, this medicine can be used in patients with moderate to severe atopic dermatitis, in people from 12 years of age weighing more than 40 kg [58,59]. The side effects of this drug are alike those of dupilumab—the most common are conjunctivitis and local reactions [78].

Tralokinumab is a monoclonal antibody with a mechanism similar to lebrikizumab—it also binds to free IL-13, but in a different epitome, preventing binding to the receptor and thus inhibiting the entire subsequent inflammatory cascade [55]. It is, like lebrikizumab, registered for use in patients 12 years of age and older [58,59]. It should therefore be remembered that the use of tralokinumab and lebrikizumab in children under 12 years of age involves a form of off-label therapy.

The second group mentioned above are Janus kinase (JAK) inhibitors. JAK inhibitors demonstrate an improved safety profile compared to traditional drugs and have proved themselves to be an effective form of therapy [39]. They inhibit the phosphorylation of intracellular tyrosine kinases, the Janus kinases (JAKs), thereby disrupting the JAK-STAT pathway [39]. This leads to the inhibition of multiple immune signal routes, involved in the pathogenesis of AD [40]. Their currently registered AD treatment representatives are upadacitinib, abrocitinib and baricitinib [62]. Upadacitinib and abrocitinib are both selective JAK1 inhibitors [62,79,80]. There is a shortage of reports of their off-label use in the current literature; however, if used in children under 12 years of age, it would refer to this form of therapy [58,59]. Another mentioned drug is baricitinib. This Janus kinase inhibitor has a registration allowing it to be marketed in Europe, but in the United States of America it would still be used off-label [62]. Its action is based on selective inhibition of JAK1 and JAK2 functions [33,40,41]. Previous studies in the treatment of AD report its effectiveness [33]. Side effects of this therapy include increased LDL cholesterol levels, upper respiratory tract infections and headaches; however, they are not very common [40,53]. According to the SmPC, baricitinib should be used in patients over 2 years of age [58,59]. One more drug being JAK inhibitor; however, slightly different from the previously mentioned ones is ruxolitinib [81]. It differs in terms of the form of administration—it is applied topically [58,59,81,82]. The clinical trials to date indicate the potential use of this medication in the treatment of AD, although currently the only dermatosis indicated in its SmPC is vitiligo in patients from 12 years of age [58,59,81]. The long-term effects of ruxolitinib are also unknown, so its role in the treatment of AD remains unclear.

It is worth mentioning that studies are being conducted on the use of nemolizumab, rocatinlimab, amlitelimab, tezepelumab, telazorlimab and many others [55,83,84]. These drugs are not yet registered in European countries, but it can be expected that at least some of them will soon appear in the arsenal of dermatologists to manage AD [40,57,73].

**Table 1:** Drugs used in the treatment of atopic dermatitis (AD) [58,59].

Drug, Concentration (%)	Registration in Treatment of AD	Age of Registration (Years)	Route of Administration	Form
Hydrocortisone 1%	yes	- *	Topical	Cream
Prednisolone 0.5%	yes	2	Topical	Cream
Flumetasone pivalate 0.02%	yes	2	Topical	Cream
Triamcinolone acetonide 0.1%	yes	3	Topical	Cream
Hydrocortisone butyrate 0.1%	yes	-	Topical	Cream
Fluticasone propionate 0.05%	yes	1	Topical	Cream, ointment
Alclometasone 0.05%	yes	1	Topical	Cream, ointment
Fluocinolone acetonide 0.025%	yes	2	Topical	Cream, ointment, gel
Methylprednisolone aceponate 0.1%	yes	2	Topical	Cream, emulsion, ointment
Mometasone furoate 0.1%	yes	2	Topical	Cream, ointment
Betamethasone dipropionate 0.05%	yes	1	Topical	Cream, ointment
Clobetasol propionate 0.05%	yes	1	Topical	Cream, ointment
Tacrolimus 0.1%	yes	16	Topical	Ointment
Tacrolimus 0.03%	yes	2	Topical	Ointment
Pimecrolimus	yes	3 months	Topical	Cream
Crisaborole	yes	2	Topical	Ointment
Methylprednisolone	yes	-	Oral/i.m./i.v.	Tablet, powder
Prednisone	yes	-	Oral	Tablet
Prednisolone	yes	-	Oral/i.m./i.v.	Tablet, powder
Hydroxyzine	no	1	Oral	Tablet, syrup
Desloratadine	no	1	Oral	Tablet, oral solution
Cetirizine	no	2	Oral	Tablet, syrup, oral drops
Levocetirizine	no	2	Oral	Tablet, oral solution
Loratadine	no	2	Oral	Tablet, syrup, soft capsules
Rupatadine	no	2	Oral	Tablet, oral solution
Fexofenadine	no	12	Oral	Tablet
Bilastine	no	6	Oral	Tablet
Montelukast	no	6 months	Oral	Tablet, granulate
Cyclosporine A	yes	18	Oral	Soft capsules, oral solution
Methotrexate	no	18	Oral/i.m./i.v./s.c	Tablets, oral solution, solution for injection
Azathioprine	no	unspecified	Oral	Tablets
Mycophenolate mofetil	no	2	Oral	Tablets, capsules, powder for oral suspension
Dupilumab	yes	6 months	S.c.	Solution for injection
Tralokinumab	yes	12	S.c.	Solution for injection
Lebrikizumab	yes	12	S.c.	Solution for injection
Upadacitinib	yes	12	Oral	Tablet
Abrocitinib	yes	12	Oral	Tablet
Baricitinib	yes	2	Oral	Tablet
Ruxolitinib	no	12	Topical	Cream

*—SmPCs indicate that there are no age restrictions for this preparation.

### 2.1. Psoriasis

Psoriasis is an immune-mediated inflammatory dermatosis [85,86,87]. It is one of the 10 most common dermatological diseases in the world, as it affects about 1–3% of the world’s population [22,88,89]. It is not possible to isolate one factor leading to its development—pathophysiology is extremely complex [86,87]. Current publications emphasize the fundamental role of immunological dysregulations in the evolution of this disease, with other components being genetic and environmental factors [86,87,90]. There are five types of this dermatosis: plaque, guttate, inverse, pustular and erythrodermic [89,90,91]. The most prevalent form is the first of the above, in which erythematous, sharply demarcated plaques with silver scales occur [87,89,92]. Diagnosis is usually made based on the findings in a physical examination [87,89]. Clinical assessment is also used to evaluate the severity of the disease, with the most commonly implemented scale being the Psoriasis Area and Severity Index (PASI), which is supplemented by the Body Surface Area (BSA) and Dermatology Life Quality (DLQI) [87]. Determining severity of psoriasis, as in the case of other inflammatory dermatoses, facilitates the selection of appropriate therapy and also allows monitoring of treatment progress [93,94].

Therapy used in psoriasis is typically divided into topical and systemic as well as phototherapy [89]. Topical drugs are usually used in mild disease [89]. These include topical steroids, vitamin D3 analogues, topical calcineurin inhibitors, coal tar, tazarotene, anthralin (dithranol) and keratolytics (Table 2) [87,89,95]. They can be supplemented with phototherapy, of which the most common are currently broadband (280–320 nm) and narrowband (311–313 nm) UVB phototherapy and PUVa photochemotherapy, which involves the use of UVA rays after sensitization with psoralens [87,89]. In the case of ineffectiveness of these forms of treatment or more advanced disease, systemic agents are implemented [89]. Drugs used systemically include methotrexate, cyclosporine, acitretin, fumaric acid esters, apremilast, as well as new biological drugs—adalimumab, certolizumab, etanercept, infliximab (anti-TNF agents), ustekinumab (anti-IL-12 and IL-23), guselkumab, risankizumab, tildrakizumab (anti-IL-23), bimekizumab, brodalumab, ixekizumab and secukinumab (anti -IL-17) [87,89,90,95]. In addition, efforts are being made to register new drugs, currently being under clinical trials, so it can be expected that the range of available substances will expand even further in the coming years [95].

Despite the emergence of novel drugs on the market, some groups suffering from discussed dermatosis are still treated off-label. Patients particularly vulnerable to therapy outside the SmPCs guidelines are minors [96]. Studies show that over 30% of children with psoriasis can be treated off-label [23]. There are many publications on this phenomenon in the literature, and yet no unified guidelines have been developed so far, which only emphasizes how important the problem is of psoriasis treatment in juveniles [96,97]. In most of them, treatment is initiated with topical preparations and usually they are sufficient to achieve a satisfactory clinical response [96]. Unfortunately, the only topical drugs registered for the treatment of psoriasis in children are glucocorticosteroids (GCSs), keratolytics and topical coal tar [98]. GCSs are marketed for use, depending on the specific preparation, from 1 or 2 years of age, keratolytics usually from 2 years of age and topical coal tar from 12 years of age [58,59]. Other preparations, including topical calcineurin inhibitors, topical retinoids, vitamin D analogues do not include childhood psoriasis in their SmPCs [97,98].

Local calcineurin inhibitors have a well-established role in managing psoriasis. Regardless of age group, they are used especially in places that are more exposed to the adverse effects of steroids, such as the face or urogenital area [96,99,100]. From a theoretical point of view, these preparations have an effect of weakening the function of T lymphocytes and the production of pro-inflammatory cytokines, which should translate into good treatment effects [101]. Previous studies have proven the effectiveness and safety of tacrolimus 0.1%, while in the case of pimecrolimus, there is still a shortage of studies proving such features of therapy with this preparation; however, available ones also indicate its potency [96,99,100]. Despite that, these drugs are still not registered for the treatment of psoriasis and, regardless of the age group, are used off-label [102].

Vitamin D3 analogues also play an important role in the topical treatment of psoriasis, regardless of the patient’s years [103]. They can be used independently or in combination with a topical steroid [101,104]. Their mechanism of action is decreasing the hyperproliferation of keratinocytes, balancing the immune response and therefore the inflammation [101,103,105]. Calcipotriene and calcitriol have been shown to be both effective and safe when used as monotherapy [104]. These features are also demonstrated by a preparation combining calcipotriol and betamethasone dipropionate [96,98]. Unfortunately, these drugs are registered for use in people over 18 years of age, which makes their use in minors an off-label therapy [58,59].

Other substances used off-label in the pediatric population are topical retinoids, including tazarotene [98]. Its action is based on limiting keratinocyte proliferation and promoting their differentiation [101]. The available literature does not allow determining its effectiveness and safety in the treatment of children suffering from psoriasis [101].

Dithranol, also known as antralin, is another drug used in pediatric psoriasis [96]. It has anti-inflammatory and antiproliferative effects, which interrupts the process leading to the development of plaques [106]. It has proven itself to be an effective and safe method of treatment and is used both in the pediatric and adult population [96,98,104].

There are also reports of the use of botulinum toxin (BoNT) in the treatment of psoriatic lesions that have been resistant to at least two other forms of treatment [107,108,109,110]. However, the results of these studies are not clear, and it is likely that the use of BoNT in treatment will be limited by practical considerations—the need for multiple injections in each affected area, and the risk of causing muscle weakness [102,104]. When it comes to systemic drugs, a significant part of them is also used off-label, especially in the pediatric population [82]. In the case of cyclosporine (CsA), for which the mechanism of action is described in Section 2.1, implementing it in people under 18 years of age exceeds the indications included in the SmPC [58,59]. However, unlike in the case of AD, the effectiveness of this drug in the treatment of pediatric psoriasis is unclear [82,99,106]. Therefore, if CsA treatment in a child is necessary, it should be as short as possible and at the lowest possible dose [82].

Methotrexate (MTX), despite its established role in adult psoriasis, is not currently registered for the treatment of this disease in children [82]. Its mode of action is also described in Section 2.1 of this publication. Studies indicate its effectiveness in its use in minors, but it should be used with caution, and folic acid supplementation should be implemented to limit side effects [82,99,106].

Fumaric acid esters, other drugs registered for use only in adults, are also sometimes applied off-label in the pediatric population [82,107]. They influence many pathogenic elements of psoriasis—they lead to decreased proliferation and activation of T lymphocytes, reduce production of pro-inflammatory cytokines and result in inhibition of dendritic cell maturation [82,108]. Unfortunately, their use may be associated with adverse reactions in the digestive system [105]. Despite the limited amount of research on their use in minors, they can be included in therapy if other forms of treatment are ineffective [82].

A drug sometimes cited in the context of off-label therapy for psoriasis is apremilast [87,101]. This medication, a phosphodiesterase-4 inhibitor, has a beneficial effect on the immune system—it inhibits pro-inflammatory reactions, leading to a decrease in the amount of cytokines such as IL-2, IL-12 and TNF-alpha, and also promotes anti-inflammatory processes, which can potentially lead to a good clinical effect of the treatment [87]. It is registered for use in the case of moderate to severe psoriasis from 18 years of age [87,101]. Recently, however, it was approved by the FDA for children aged 6 and above; therefore, changes in registration can also be expected in European legislation in the near future.

A new category of medications used in managing psoriasis are biological drugs [87]. Currently, many preparations belonging to this group are available, with a significant number of them also registered for the treatment of pediatric psoriasis [111,112,113,114,115]. The medicines that, according to the SmPCs, can be used in children are adalimumab, registered from 4 years of age, as well as etanercept, ustekinumab, ixekizumab and secukinumab registered by EMA from 6 years of age [107] Other biologics, if implemented in minors, would be applied in an off-label manner. Available publications do not directly address the issue of their use outside the registration, although there are some reports assessing their effectiveness and safety in pediatric populations [114]. Certolizumab and infliximab, which are TNF-alpha inhibitors, are not often included in publications in the context of treatment of childhood psoriasis [115]. In turn, efficacy and safety in the pediatric population were assessed in clinical trials of anti-IL-23 agents: NCT03451851 for guselkumab, NCT04435600 for risankizumab and NCT03997786 for tildrakizumab, although this did not lead to their registration being expanded [114]. In the case of the interleukin 17 inhibitor, brodalumab, a study evaluating the potency of this drug in managing pediatric psoriasis, compared to ustekinumab, was published [116]. It showed its efficacy and safety in treating children with the discussed dermatosis [116].

Use outside the SmPCs guidelines also includes recommending biologicals in types other than registered psoriasis. An example of such an application is applying golimumab in a patient with erythrodermic psoriasis [117]. In the cited case report, the use in another type of dermatosis than indicated by SmPC brought the desired therapeutic effect. However, the lack of larger studies prevents one from determining the role of this drug in the treatment of erythrodermic psoriasis. There are also reports in the literature about the use of secukinumab outside the registered indications [118,119]. They concern the use of this drug in pustular psoriasis, as well as in a higher than recommended dosage [118,119]. Data on the effectiveness of these therapies are limited; in the case of pustular psoriasis, the study group consisted of four people, while in the case of increased dosage, one case report and one study on 25 people were described [118,119,120]. Because of limited group sizes, despite the observation of remission of lesions, the role of this drug in the treatment not included in SmPC is still to be established [118,119,120]. The use of a different dose than indicated in the product characteristics is not limited to secukinumab. A study published in 2015 indicated that dose regimens changes are a common occurrence in clinical practice [121].

A medication that may fit into the classic off-label understanding, i.e., used in a disease other than the one indicated in the SmPC, is mirikizumab [122]. This anti-IL-23 agent is currently registered only in managing ulcerative colitis [58,59]. However, there are randomized studies that indicate its effectiveness in the treatment of psoriasis, and also prove that the safety profile of mirikizumab is consistent with other IL-23 inhibitors, already approved for the treatment of the discussed dermatosis [122].

Publications also raise the topic of off-label use of Janus kinase inhibitors [123]. One of the discussed drugs is ruxolitinib [124]. This topical JAK-1 and 2 inhibitor has been shown to be effective in reducing PASI in adults in randomized clinical trials to date [124]. Another Janus kinase inhibitor acting on JAK1/3—tofacitinib—has also undergone RCTs for both its topical and systemic forms [125]. Most of them have shown the effectiveness and safety of this drug in adults with plaque psoriasis [125]. Different medication, cited in the context of therapy outside the registration, is baricitinib [126]. As mentioned in Section 2.1, it inhibits JAK1 and JAK2, which leads to the silencing of the inflammatory response [127]. So far, its SmPC includes several diseases, but not psoriasis [128]. Some studies indicate the efficacy of this drug in reducing PASI, as well as the safety of this therapy, although more recent publications report its insufficient effectiveness in the treatment of discussed dermatosis [127,128].

The list of drugs used off-label in psoriasis is not limited to those listed above, as publications also mention the use of crisaborole, oral isotretinoin, naltrexone, antihistamines or sulfasalazine [129,130,131,132,133]. However, these are not common therapies and the available data are sparse, making it difficult to assess their effectiveness and safety [129,130,131,132,133].

### 2.2. Acne Vulgaris

Acne vulgaris is an inflammatory dermatosis that can affect almost 100% of people at some point in their lives [134]. It most often occurs in teenagers, although it is not limited to this age group, as it can persist into adulthood [134,135,136,137,138]. As with other skin diseases discussed in this review, acne has a complex pathogenesis [134]. The basis for the development of this dermatosis is most often given as overproduction of sebum and abnormal keratinization, especially in the area of hair follicle openings, colonization of cutibacterium acnes and the inflammatory reaction stimulated by them [134,135,137,138]. These complex processes lead to the formation of microcomedones, which can then transform into a non-inflammatory eruption—an open or closed comedone or into an inflammatory ones—papules, pustules and nodules [134,135,136,139]. There are several forms of acne, depending on which type of eruptions predominates: comedone, papulopustular, purulent and fulminant [134]. Regardless of the type, this dermatosis should be recognized and treated [134]. It significantly affects the self-esteem of patients and is associated with a more frequent occurrence of depression, anxiety, psychosomatic symptoms or even suicidal ideation [134,139].

Acne treatment can be applied topically or systemically, and different forms of therapy are often combined (Table 3) [134,137]. Local retinoids, benzoyl peroxide, antibiotics and azelaic acid are primarily used [134,137,138,140]. These drugs have comedolytic, antibacterial and anti-inflammatory effects, acting multi-directionally on the pathogenesis of acne [134].

Although discussed dermatosis is most common in teenagers, it sometimes appears in children or continues into adulthood [134]. Especially in those under 12 years of age, topical treatment often goes beyond the scope of the SmPCs [141]. Publications indicate that the most commonly prescribed off-label preparations in preadolescents are topical retinoids, benzoyl peroxide and topical antibiotics, frequently in the form of combination preparations [141,142]. Typically, these forms of treatment are effective and side effects are limited, even among young patients [141].

Sometimes, however, topical preparations are not sufficient [134,138]. The most classic medication used in this case is isotretinoin [134,135,143]. Its mechanism of action is based on binding to RAR and RXR receptors, the activation of which, by influencing gene transcription, leads to the regulation of the immune system, including IL-2 and IFN-gamma, reduced sebum production, calmed hair follicle keratinization and decreased number of Cutibacterium acnes bacteria [141,142,143,144,145,146]. It therefore influences four main elements of acne pathogenesis. Many studies have shown both the safety and effectiveness of this drug in the treatment of acne vulgaris [134,143]. However, it is important to remember the limitations of this drug—first of all, it is a teratogenic substance; therefore, the condition for initiating treatment is use of an effective form of contraception during and one month after the end of therapy by the patient [58,59,134]. In addition, during treatment, side effects may occur such as dryness of skin and mucous membranes, cheilitis, nose bleeds or hyperlipidemia [58,59,134]. In compliance with the SmPC, isotretinoin should be used in patients with severe forms of acne, at a dose of 0.5 to 1 mg/kg of body weight daily with a total dose of 120 to 150 mg/kg per treatment cycle [58,59,143,147]. According to the current literature, isotretinoin is also applied in other forms of discussed dermatosis and in other dosing regimens than those indicated in the product characteristics. An example of such off-label use is the recommendation of isotretinoin in a low dose, usually between 0.1 and 0.3 mg/kg body weight [139,143,148]. There is no clear conclusion as to whether this dosing method is effective, although some studies demonstrated that reducing the dose of isotretinoin is associated with a higher risk of relapse, but less frequent side effects [139,143,148,149]. Nevertheless, there is a lack of RCTs involving large groups of study participants, analyzing whether maintaining the cumulative dose, despite the reduction in the daily dose, would allow maintaining the efficacy of this drug [143,150]. Another mentioned off-label example is use in different forms of acne. There are many reports of isotretinoin being successfully applied in mild and moderate cases [139,147,151]. This is often associated with the previously mentioned form of treatment outside the SmPC guidelines; i.e., at a reduced dose [139,147,151,152].

Other drugs used systemically include antibiotics [134]. While tetracycline or doxycycline include in their SmPCs treatment of acne vulgaris, many antibiotics, despite not being registered for the treatment of acne vulgaris, are used in clinical practice [146,153]. However, new therapeutic options are appearing on the market—a new type of tetracycline, sarecycline, is already registered by the FDA, but still has not received EMA approval [154,155]. Like other antibiotics used to treat this dermatosis, it has a bacteriostatic effect on Gram-negative bacteria, including Cutibacterium acnes, which, by promoting inflammation in the hair follicles, contributes to the development of acne vulgaris [156]. Its advantage is less interference with the intestinal microbiome, with maintained comparable effectiveness, as well as a different dosing regimen—it should be taken only once a day [156]. This raises the hopes for reducing antibiotic use outside the SmPCs.

Further forms of off-label treatment, which can be implemented in women with acne vulgaris, are hormone-regulating therapies [157]. They include, among others, spironolactone [152,153]. This aldosterone receptor antagonist allows for the elimination of excessive stimulation of the sebaceous glands by androgens, which may occur in females with the discussed dermatosis [158,159,160]. A 2024 RCT demonstrated the higher effectiveness of spironolactone in the treatment of moderate acne in adult women, compared to doxycycline, as well as its favorable profile in terms of side effects [160]. It may however be associated with symptoms such as decreased libido, menstrual irregularities, or breast tenderness, and should not be used by pregnant females [157,161].

Combined oral contraceptives (COCs) are another example of hormone-modulating therapy, relatively often implemented in female acne [134,157]. These preparations consist of estrogen, usually ethinylestradiol, and progestogens [157]. The action of the former, results in the inhibition of ovarian and adrenal androgen production, by increasing the creation of sex hormone-binding globulin (SHBG), which in turn leads to a decrease in free testosterone levels and reduction of stimulation of androgen receptors [157,162]. The progestogen component may have different effects, depending on the generation of the specific substance, but overall COCs have an antiandrogenic effect [151,153,157]. As with spironolactone, this allows the elimination of excessive follicular keratosis and decreasing the stimulation of sebum production [153,157,162]. This translates into the effectiveness of these preparations in the treatment of acne [153,156,157]. Unfortunately, their use can be associated with serious complications, such as thromboembolism and pulmonary embolism [157,163]. For this reason, before prescribing oral contraceptives, the patient’s individual thromboembolic risk should be assessed and the balance of profits and losses should be considered [157]. The question of whether the use of COCs in the treatment of acne is an off-label treatment is controversial. There are preparations that have registrations in the treatment of this dermatosis in patients, who want to achieve a contraceptive effect, but physicians usually do not limit themselves to those expressing such a desire [151,153,158]. However, it is difficult to determine whether this is an off-label use, because although the contraceptive result may play a secondary role, the condition included in SmPCs, i.e., the wish to achieve such an effect, is still met.

There are also some reports of other drugs used outside the registrations to manage acne vulgaris: metformin, adalimumab, infliximab or etanercept are some of them [164,165]. Nevertheless, the mentioned biological drugs can also lead to the development of skin lesions instead of their quieting, so their role in managing acne is still unclear [166]. The literature also refers to the issue of therapies not limited to pharmacological forms and as examples of off-label treatment gives, among others, intense pulsed light (IPL) therapy or photodynamic therapy [167,168,169]. Both of these forms of treatment may be effective in some acne patients, according to current research [169].

### 2.3. Rosacea

Rosacea is a chronic inflammatory skin disease [170]. The pathogenesis of this dermatosis is not yet fully understood, but genetic predispositions, immune dysregulation, mediated by mast cells, IL-37 and kallikrein 5, as well as neurovascular abnormalities, *Demodex* spp. or *Staphylococcus epidermidis* infestation, play a significant role [170,171,172]. It manifests itself, depending on the form, with persistent skin erythema and telangiectasia (erythematous-vascular form), papules and pustules (papulopustular form), thickening of the skin and raised nodular changes, especially in the nose, which is called rhinophyma (phymatous form), redness of the eyelids, blurred vision and a feeling of sand in the eye (ocular form) [170,171,173]. This disease most often affects people aged 30 to 50 years old and is generally more common among fair-skin women, although the phymatous form is more prevalent in men [174,175,176]. Treatment of rosacea is long-term, due to the chronic and recurrent character of this dermatosis. The basis of the therapy is proper skin care and sun protection, which are accompanied by local or systemic treatment (Table 4) [165,167,177,178]. Locally applied drugs are antibiotics—especially metronidazole, but also ivermectin, azelaic acid and brimonidine tartrate [178]. In turn, systemic therapy includes antibiotics, usually doxycycline or tetracycline, as well as isotretinoin, which is considered to be off-label use [178]. In addition, procedures such as an intensive pulsed light (IPL), laser therapy, CO_2_ laser and electroresection are used, especially in the case of phymatous rosacea [178].

Off-label treatment includes among others antibiotics, both applied topically, such as 2% erythromycin, and those in systemic form, such as erythromycin, minocycline and azithromycin [22,174,179]. Their antibacterial and anti-inflammatory effects improve the condition of skin in people with rosacea [165]. Topical preparations can cause irritation and dry skin, while systemic ones can sometimes cause more serious side effects, such as gastrointestinal upset, headaches or allergic reactions [165].

Topical retinoids are one more example of treatment outside the SmPC framework. Among others, tretinoin is used, in the form of a 0.025% cream or lotion or in the form of a 0.01% gel [165]. Its principle of action is similar to that in acne vulgaris—its anti-inflammatory, antibacterial and keratinization regulating effects are also used in rosacea [165]. Additionally, in the event of this dermatosis, it is necessary to remember the side effects—local irritation, photosensitivity and the contraindication on applying in pregnant patients are limitations to the use of this treatment method [165].

Combination preparations can also be used. A representative case is a preparation combining 1.2% clindamycin and 0.025% tretinoin in a gel [169]. Previous studies suggest that it may be effective in treating erythematous-vascular forms, but it is unlikely to provide clinical improvement in people with the papulopustular form [169,170].

A different treatment method implemented in an off-label manner is the use of permethrin 5% gel [171]. RCTs have shown significant improvement in the clinical picture in patients applying this drug, which works mainly by reducing the number of Demodex folliculorum [171]. It is achieved by paralyzing the nervous system of these parasites, which is performed by disrupting the functioning of sodium transport across nerve cell membranes. Due to its selective action on arthropods, this preparation has a favorable safety profile [172].

Another group of drugs used off-label in the treatment of rosacea are topical calcineurin inhibitors (TCI). As in the case of other dermatoses, in the event of this skin disease, TCIs also act mainly through their immunomodulatory effect, leading to reduced production of proinflammatory cytokines [40]. Tacrolimus ointment, which belongs to this group, has been shown to be effective in treating erythema, while it had no effect on papular changes [173]. However, this clinical trial included a very limited number of participants, so to draw correct conclusions, the study should be repeated on a larger number of patients [173]. Limited data also apply to pimecrolimus, which according to one study, demonstrates efficacy similar to that of topical metronidazole, while following another publication its therapeutic effects after 4 weeks do not differ from placebo [40].

A different example of a drug used outside the SmPC framework is sodium sulfacetamide 10% with or without 5% sulfur cream or lotion. This drug is already registered by the FDA for the treatment of rosacea, but according to EMA guidelines its use is limited to the management of acne vulgaris [174]. Studies to date indicate the effectiveness of this preparation, and adverse effects are usually limited to local reactions [174]. Its action, like many of the previous ones, is based on the antibacterial and keratolytic effect, although sometimes the addition of, for example, 10% urea to this preparation also allows for achieving better skin hydration [175].

Isotretinoin is one more, non-negligible drug used off-label in the management of rosacea [136]. Its action is based on anti-inflammatory effects, regulating the function of sebaceous glands and decreasing the blood flow through the skin [122,136,176]. The side effects described in Section 2.3 may constitute an important limitation to the use of this preparation. It should also be remembered that relapses occur more often in the treatment of rosacea than in the treatment of acne vulgaris, being 45–58.3% within a year [135,136]. Despite that, both full and low doses of isotretinoin have proven to be an effective method in managing several forms, with the most efficient dose appearing to be 0.3 mg/kg/day for 12 weeks of treatment [136,176,179,180].

A relatively novel therapeutic option is applying the botulinum toxin A (BoNT-A) [181,182]. Its action in rosacea is likely based on blocking the secretion of acetylcholine, which leads to reduced cutaneous vasodilation [102,182,183]. The use of multiple injections with the administration of small amounts of this preparation has shown to be effective in the treatment of facial erythema, which is the basic symptom of the discussed dermatosis [181,182,183]. Research conducted to date also suggests the safety of this preparation [182]. However, such use is not covered by SmPC of this product [182].

## 3. Conclusions

Off-label therapy is widespread in the treatment of inflammatory dermatoses such as atopic dermatitis, psoriasis, acne vulgaris and rosacea. The efficacy of used drugs vary—some treatment methods outside the SmPC guidelines have a well-established position in the therapy of a given disease entity, supported by adequate clinical trials, but unfortunately this does not apply to all analyzed preparations. For most dermatoses, there is no data on what percentage of patients are treated off-label and how often specific preparations outside of SmPC are used. Further studies on the mechanisms of action, effectiveness and safety of the drugs discussed would allow for more evidence-based medicine treatment of those suffering from inflammatory skin diseases. In addition, new research on the prevalence of use would contribute novel knowledge and improve our understanding of the discussed approach. It is undeniable that, in certain situations, off-label treatment may offer the most optimal solution. It can be less risky than medical experiments and more adaptable to patient needs than strictly following SmPC guidelines. Additionally, this approach can sometimes lead to broader drug approvals in the future. Nonetheless, increasing awareness of treatments beyond SmPCs through further research could not only enhance the medical community’s understanding of this practice but also offer patients more informed and evidence-based care.

## Figures and Tables

**Table 2:** Drugs used in the treatment of psoriasis [58,59].

Drug, Concentration (%)	Registration in Treatment of Psoriasis	Type of Psoriasis Included in SmPCs	Age of Registration (Years)	Route of Administration	Form
Hydrocortisone 1%	yes	All	- *	Topical	Cream
Prednisolone 0.5%	yes	All	2	Topical	Cream
Flumetasone pivalate 0.02%	yes	All	2	Topical	Cream
Triamcinolone acetonide 0.1%	yes	All	3	Topical	Cream
Hydrocortisone butyrate 0.1%	yes	All	-	Topical	Cream
Fluticasone propionate 0.05%	yes	All	1	Topical	Cream, ointment
Alclometasone 0.05%	yes	All	1	Topical	Cream, ointment
Fluocinolone acetonide 0.025%	yes	All	2	Topical	Cream, ointment, gel
Methylprednisolone aceponate 0.1%	yes	All	2	Topical	Cream, emulsion, ointment
Mometasone furoate 0.1%	yes	All	2	Topical	Cream, ointment
Betamethasone dipropionate 0.05%	yes	All	1	Topical	Cream, ointment
Clobetasol propionate 0.05%	yes	All	1	Topical	Cream, ointment
Tacrolimus 0.1%	no	None	16	Topical	Ointment
Tacrolimus 0.03%	no	None	2	Topical	Ointment
Pimecrolimus	no	None	3 months	Topical	Cream
Calcipotriol	yes	Psoriasis vulgaris, Scalp psoriasis	18	Topical	Ointment, gel, cutaneous solution
Tacalcitol	yes	Psoriasis vulgaris	18	Topical	Ointment
Coal tar	yes	All	12	Topical	Cutaneous emulsion, solution, cream
Tazarotene	yes	Psoriasis vulgaris	18	Topical	Gel
Anthralin (dithranol)	yes	All	-	Topical	Cream
Salicylic acid 3–10%	yes	All	-	Topical	Ointment, cream, topical solution
Urea	yes	All	-	Topical	Ointment, cream
Botulinum toxin	no	None	12	i.m.	Solution for injection
Methotrexate	yes	Severe psoriasis	18	Oral/i.m./i.v./s.c.	Tablet, solution for injection
Cyclosporine A	yes	Severe psoriasis	18	Oral	Soft capsules, oral solution
Acitretin	yes	Severe psoriasis, especially erythrodermic and pustular	-	Oral	Capsules
Fumaric acid esters (diethyl fumarate)	yes	Moderate plaque psoriasis	18	Oral	Gastro- resistant tablet
Apremilast	yes	Moderate to severe plaque psoriasis	18	Oral	Tablet
Adalimumab	yes	Severe chronic plaque psoriasis	4	sc.	Solution for injection
Certolizumab	yes	Moderate to severe plaque psoriasis	18	sc.	Solution for injection
Etanercept	yes	Moderate to severe plaque psoriasis	6	sc.	Solution for injection
Infliximab	yes	Moderate to severe plaque psoriasis	18	i.v.	Solution for injection
Ustekinumab	yes	Moderate to severe plaque psoriasis	6	sc.	Solution for injection
Guselkumab	yes	Moderate to severe plaque psoriasis	18	sc.	Solution for injection
Risankizumab	yes	Moderate to severe plaque psoriasis	18	sc.	Solution for injection
Tildrakizumab	yes	Moderate to severe plaque psoriasis	18	sc.	Solution for injection
Bimekizumab	yes	Moderate to severe plaque psoriasis	18	sc.	Solution for injection
Brodalumab	yes	Moderate to severe plaque psoriasis	18	sc.	Solution for injection
Ixekizumab	yes	Moderate to severe plaque psoriasis	6	sc.	Solution for injection
Secukinumab	yes	Moderate to severe plaque psoriasis	6	sc.	Solution for injection
Mirikizumab	no	None	18	i.v.	Solution for injection

*—SmPCs indicate that there are no age restrictions for this preparation.

**Table 3:** Drugs used in the treatment of acne vulgaris [58,59].

Drug, Concentration (%)	Registration in Treatment of Acne Vulgaris	Type of Acne Included in SmPCs	Age of Registration (Years)	Route of Administration	Form
Adapalene, 0.1%	yes	Mild to moderate acne vulgaris, comedone acne, papulopustular acne	12	Topical	Cream, gel
Isotretinoin, 0.05%	yes	Mild to moderate acne vulgaris	12	Topical	Gel
Tretinoin, 0.05% and 0.1%	yes	Acne vulgaris	12	Topical	Cream, gel
Trifarotene, 0.05%	yes	Acne vulgaris	12	Topical	Cream
Tazarotene, 0.1%	yes	Mild to moderate acne vulgaris	12	Topical	Gel
Benzoyl peroxide, 5%	yes	Acne vulgaris	12	Topical	Gel, cream
Clindamycin	yes	Mild to moderate acne vulgaris	12	Topical	Solution, gel
Erythromycin, 2%	yes	Acne vulgaris	- *	Topical	Solution, gel
Azelaic acid	yes	Mild to moderate acne vulgaris, papulopustular acne	12	Topical	Gel
Isotretinoin	yes	Severe acne	12	Oral	Soft capsules
Doxycycline	yes	Acne vulgaris	12	Oral	Tablet, capsule
Lymecycline	yes	All	12	Oral	Capsule
Minocycline	yes	All	12	Oral	Tablet, capsule
Erythromycin	yes	Pustular acne	2	Oral	Tablet
Trimetoprim	no	None	6	Oral	Tablet
Cotrimoxazole	no	None	12	Oral	Tablet
Spironolactone	no	None	Unspecified	Oral	Tablet
Combined oral contraceptives	yes	Mild to moderate acne vulgaris in women wishing to achieve contraceptive effect	In menstruating patients	Oral	Tablet
Metformin	no	None	10	Oral	Tablet

*—SmPCs indicate that there are no age restrictions for this preparation.

**Table 4:** Drugs used in the treatment of rosacea [58,59].

Drug, Concentration (%)	Registration in Treatment of Rosacea	Age of Registration (Years)	Route of Administration	Form
Adapalene, 0.1%	no	12	Topical	Cream, gel
Isotretinoin, 0.05%	no	12	Topical	Gel
Tretinoin, 0.05% and 0.1%	no	12	Topical	Cream, gel
Trifarotene, 0.05%	no	12	Topical	Cream
Tazarotene, 0.1%	no	12	Topical	Gel
Tacrolimus 0.1%	no	16	Topical	Ointment
Tacrolimus 0.03%	no	2	Topical	Ointment
Pimecrolimus	no	3 months	Topical	Ointment
Azelaic acid	yes	12	Topical	Gel
Brimonidine tartrate	yes	18	Topical	Gel
Erythromycin, 2%	no	- *	Topical	Solution, gel
Metronidazole, 0.75%	yes	18	Topical	Gel
Permethrin, 5%	no	2 months	Topical	Cream
Ivermectin	yes	18	Topical	Cream
Sodium sulfacetamide, 10%	no	unspecified	Topical	Cream, lotion
Sodium sulfacetamide 10% with sulfur 3%	no	unspecified	Topical	Cream, lotion
Isotretinoin	no	12	Oral	Soft capsules
Doxycycline	no	12	Oral	Tablet, capsule
Limecycline	yes	12	Oral	Capsule
Minocycline	no	12	Oral	Tablet, capsule
Erythromycin	no	2	Oral	Tablet
Azithromycin	no	unspecified	Oral	Tablet
Botulinum toxin	no	12	im.	Solution for injection

*—SmPCs indicate that there are no age restrictions for this preparation.

## Abbreviations

The following abbreviations are used in this manuscript:

AD	Atopic dermatitis
AZA	Azathioprine
BoNT-A	Botulinum toxin A
BSA	Body Surface Area
COC	Combined oral contraceptives
CsA	Cyclosporine
DLQI	Dermatology Life Quality
EC	European Committee
EMA	European Medicines Agencies
FDA	Food and Drug Administration
GCS	Glucocorticosteroids
GM-CSF	Granulocyte-macrophage colony-stimulating factor
HLA	Human leukocyte antigen
i.m.	Intramuscularis
i.v.	Intravenosa
IFN	Interferone
IL	Interleukin
IPL	Intense pulsed light
JAK	Januse
MMF	Mycophenolate mofetil
MTX	Methotrexate
PASI	Psoriasis Area and Severity Index
RCT	Randomized Clinical Trials
sc.	Subcutanea
SCORAD	Scoring Atopic Dermatitis
SmPC	Summary of Product Characteristics
TCI	Topical Calcineurin Inhibitors
TNF	Tumor necrosis factor
